# Rapamycin Induces Phenotypic Alterations in Oral Cancer Cells That May Facilitate Antitumor T Cell Responses

**DOI:** 10.3390/biomedicines12051078

**Published:** 2024-05-13

**Authors:** Amirmoezz Yonesi, Kei Tomihara, Danki Takatsuka, Hidetake Tachinami, Manabu Yamazaki, Amir Reza Younesi Jadidi, Mayu Takaichi, Shuichi Imaue, Kumiko Fujiwara, Shin-Ichi Yamada, Jun-Ichi Tanuma, Makoto Noguchi

**Affiliations:** 1 Department of Oral and Maxillofacial Surgery, Faculty of Medicine, Academic Assembly, University of Toyama, Toyama 930-0194, Japanamiryounesi.younesi@yahoo.com (A.R.Y.J.); mnoguchi@med.u-toyama.ac.jp (M.N.); 2 Division of Oral and Maxillofacial Surgery, Faculty of Dentistry, Graduate School of Medical and Dental Sciences, Niigata University, Niigata 951-8514, Japan; 3 Division of Oral Pathology, Faculty of Dentistry, Graduate School of Medical and Dental Sciences, Niigata University, Niigata 951-8514, Japantanuma@dent.niigata-u.ac.jp (J.-I.T.); 4 Department of Dentistry and Oral Surgery, Osaka Medical and Pharmaceutical University, Takatsuki 569-8686, Japan; kumiko.fujiwara@ompu.ac.jp

**Keywords:** mTOR, rapamycin, immune checkpoint inhibitor, immunomodulation, immunotherapy, oral squamous cell carcinoma

## Abstract

Objectives: In this study, we investigated the antitumor immunomodulatory effects of rapamycin in oral cancer. Study Design: We examined the proliferation, apoptosis, and migration of cancer cells and investigated the cell surface expression levels of immune accessory molecules and T cell immune responses in vitro. We investigated the effect of in vivo administration of rapamycin on immune cell distribution and T cell immune responses in oral tumor-bearing mice. Results: Rapamycin treatment significantly inhibited OSCC cell proliferation and migration, increased apoptotic cell death, and upregulated cell surface expression of several immune accessory and adhesion molecules, including CD40, CD83, PD-L1, PD-L2, MHC class I, P-selectin, and VCAM-1. These cancer cells augmented T cell proliferation. In vivo rapamycin administration significantly attenuated mouse tumor growth with an increased proportion of immune cells, including CD4^+^ T cells, CD8^+^ T cells, and dendritic cells (DCs); decreased the proportion of immune suppressive cells, such as myeloid-derived suppressor cells and regulatory T cells; enhanced DC maturation and upregulated the surface expression of CD40, CD86, and ICAM-1. Conclusions: Our results suggest that the therapeutic effect of mTOR inhibition in oral cancer can cause direct antitumor and immunomodulatory effects.

## 1. Introduction

Oral cancer is among the most common malignant tumors worldwide and oral squamous cell carcinomas (OSCCs) account for 90% of oral cancer diagnoses [1]. The treatment strategies for oral cancer include single-modality therapies—surgery, radiotherapy—or various combinations of these modalities, with or without systemic chemotherapy [2]. Although recent advancements in oral cancer treatment and diagnostic techniques have significantly improved patient outcomes, treatment strategies for high-grade tumors that are refractory to chemotherapy and radiotherapy, or with high metastatic potential, remain unknown [3].

Recently, a strategy targeting the epidermal growth factor receptor (EGFR), which is overexpressed and contributes to the aggressive behavior of oral cancer, has shown beneficial results, particularly in advanced or recurrent cases where other therapies are no longer effective [4]. Cancer immunotherapies based on targeting immune checkpoint molecules, including CTLA-4, PD-1, and PD-L1, have been clinically successful and demonstrated to improve clinical outcomes in patients with recurrent or metastatic cancer [5]; however, primary or acquired resistance to both therapies is common, and no treatment options are available to date. Therefore, novel strategies that target other specific molecules are required.

Among the dysregulation of multiple cell signaling cascades, the constitutive activation of the phosphatidylinositol 3-kinase/Akt (PI3K/Akt) signaling axis and its downstream target molecule, the mechanistic target of rapamycin (mTOR), is commonly observed in many cancers [6].

The mTOR signaling pathway is critical in tumor progression through proliferation, migration, angiogenesis, glycolytic metabolism, and lipid metabolism [7]. Therefore, mTOR signaling has been regarded as a promising target in cancer therapy. 

The constitutive activation of PI3K/Akt and mTOR signaling has also been observed in more than 30% of patients with head and neck squamous cell carcinomas [8]. Moreover, several studies have demonstrated the antitumor effect of modulating mTOR signaling using natural and synthetic inhibitors, indicating that the Akt/mTOR pathway may be a promising therapeutic target for oral cancer [9,10].

Several mTOR inhibitors have already been developed and many preclinical and clinical studies have corroborated the importance of mTOR inhibitors in cancer treatment [11,12]. Rapamycin, a pharmacological mTOR inhibitor used as an immunosuppressive drug in transplantation, has also been widely evaluated for the treatment of many cancers [13].

Although rapamycin has been demonstrated to modulate cellular processes in cancers, including proliferation, survival, angiogenesis, invasion, metastasis, autophagy, transition from epithelial to mesenchymal neoplasm, and resistance to chemotherapy or radiotherapy, its immunomodulatory effects on the tumor microenvironment remain unclear.

In this study, we examined the immunomodulatory effects of mTOR inhibitors on oral cancer via an analysis of both human OSCC cell lines and an oral cancer mouse model.

## 2. Materials and Methods

### 2.1. Tissue Samples

Twenty-seven patients (15 males, 12 females) were included in this study. The patients underwent surgical treatment for OSCC at the Department of Oral and Maxillofacial Surgery, Toyama University Hospital. The primary carcinoma sites were the tongue (*n* = 10), mandibular gingiva (*n* = 7), maxillary gingiva (*n* = 5), buccal mucosa (*n* = 3), and floor of the mouth (*n* = 2). Five patients were diagnosed with stage I primary OSCC, eight with stage II, seven with stage III, and seven with stage IV, based on the UICC TNM classification criteria for oral cavity cancer (Table 1). Tissue specimens obtained via biopsy or surgical resection were fixed in 10% formalin and embedded in paraffin. This study was approved by the ethics committee of Toyama University Hospital (R2022002). 

### 2.2. Immunohistochemistry

Paraffin sections were cut at a 4 μm thickness and prepared for further analysis by hematoxylin–eosin and immunohistochemical staining. The sections were deparaffinized, rehydrated, and then autoclaved in citric acid buffer (pH 6.0) for 10 min at 121 °C for antigen retrieval. The sections were immersed in 3% hydrogen peroxide in deionized water for 15 min at room temperature. After washing with phosphate-buffered saline (PBS), the sections were incubated with a blocking agent (Blocking One, Nacalai Tesque Inc., Kyoto, Japan) for 10 min at room temperature and reacted with rabbit monoclonal antibody against mTOR (clone 7C10, cat. #2983S, Cell Signaling Technology, Danvers, MA, USA) at a dilution of 1:250 overnight at 4 °C. The sections were washed with PBS and then incubated with horseradish peroxidase-conjugated goat anti-rabbit IgG antibody (cat #424144, Nichirei Bioscience Inc., Tokyo, Japan) for 60 min at room temperature. Peroxidase reactions were developed with a 3,3′-diaminobenzidine substrate, and the sections were counterstained with hematoxylin.

The staining intensity (0, 1+, 2+, or 3+) for each sample was determined in the hot spot area, which represented the zone with the highest number of mTOR-positive stained cells among at least 2000 carcinoma cells. The percentage of cells at each staining intensity level was calculated and the H score was derived using the following formula: (1 × % cells 1+) + (2 × % cells 2+) + (3 × % cells 3+). 

### 2.3. Human and Mouse Oral Squamous Cell Carcinoma Cell Lines

Human OSCC cell lines HSC-2, HSC-3, and Ca 9-22 were obtained from the RIKEN Bio Resource Center Cell Bank. These cells were cultured in RPMI 1640 (Thermo Fisher Scientific, Waltham, MA, USA) medium supplemented with 10% fetal bovine serum (FBS; GIBCO, NY, USA) containing 1% penicillin–streptomycin (Sigma-Aldrich, St Louis, MO, USA) and 1% sodium pyruvate (Wako, Osaka, Japan) in a wet carbon dioxide (CO_2_) gas incubator set at 5% CO_2_.

The mouse OSCC cell line NR-S1K was derived from the NR-S1 cell line, which in turn was established from C3H/HeN mice [14]. Cells were maintained in RPMI1640 medium supplemented with 10% FBS. 

### 2.4. Cell Proliferation Assay

OSCC cells were seeded into 96-well plates at a density of 1.5 × 10^4^ cells/well and cultured in the presence or absence of various concentrations of rapamycin (LC Laboratories, Woburn, MA, USA) for 72 h. The viability of the adherent cells was measured via addition of a tetrazolium salt and 4-[3-(4-iodophenyl)-2-(4-nitrophenyl)-2H-5-tetrazolio]-1,3-benzene disulfonate (WST-1) premix (Takara Bio, Kusatsu, Shiga, Japan) to each well. The cleavage of WST-1 into formazan by the metabolically active cells was measured via scanning the plates at 450 nm using a microtiter plate reader (Molecular Devices, Sunnyvale, CA, USA). 

### 2.5. Migration Assay

OSCC cells were cultured in the presence or absence of 15 nM rapamycin for 48 h, and then cultured using a CytoSelect™ 96-Well Cell Migration and Invasion Assay Kit (8 μm pore size; Cell Biolabs, Inc., San Diego, CA, USA) at a cell concentration of 1.0 × 10^4^ cells/well. After 24 h, the cells were fluorescently stained and absorbance was measured using a microtiter plate reader (Filter Max F5, Molecular Devices, Tokyo, Japan).

### 2.6. Assessment of Cellular Apoptosis

Cellular apoptosis was assessed as described previously [15]. Apoptotic cells were detected using Annexin V (BD Pharmingen, San Diego, CA, USA) staining as per manufacturer’s instructions. 

### 2.7. Mice and Tumor Model

The tumor model was made as previously described [16,17]. Female C3H/HeN mice (7–8 weeks old) were purchased from Sankyo Labo Service Corporation, Inc., and housed under specific pathogen-free conditions according to the institutional guidelines at the University of Toyama. Animal protocols were reviewed and approved by the Institutional Animal Care and Use Committee of the University of Toyama. 

NR-S1K cells (1 × 10^6^) were subcutaneously inoculated into the right masseter of C3H/HeN mice. The tumor area (length × width) was measured every three days using a caliper. This animal experiment was carried out in accordance with the ARRIVE guidelines, the Act on Welfare and Management of Animals, and the recommendations in the Guidelines for Proper Conduct of Animal Experiments of the Science Council of Japan (SCJ).

### 2.8. In Vivo Rapamycin Treatment

After 14 days (when the tumor surface area was approximately 50 mm^2^) of NR-S1K inoculation, mice were injected intraperitoneally (i.p.) with a dose of 2 mg/kg of rapamycin. Control mice were injected with saline. Seventy-two hours after the rapamycin injection, the mice were humanely sacrificed, and cells from the tumors, peripheral blood, lymph nodes, and spleens were analyzed using flow cytometry. To monitor tumor size progression, rapamycin was administered every two days after the first i.p. injection and the tumor size (length × width) was determined at 3-day intervals. 

### 2.9. Tumor Dissociation

A total of 0.02 mg/mL DNase I (Roche, Switzerland) and 1 mg/mL collagenase type IV (Sigma–Aldrich, St Louis, MO, USA) were used to digest the harvested tumors at 37 °C for 60 min. Single cell suspension was generated by filtration through a 100 μm nylon cell strainer (Corning, Glendale, AZ, USA).

### 2.10. Antibodies and Reagents

The following antibodies were obtained from Thermo Fisher Scientific: FITC-conjugated antibodies against mouse CD40, CD54, and CD83; PE-conjugated antibodies against mouse CD80, CD86, CD274, MHC class I, and MHC class II; PerCP-Cy5.5-conjugated antibodies against mouse CD4 and CD11b; PE-Cy7-conjugated antibodies against mouse CD8 and CD11c; APC-conjugated antibodies against mouse CD3e and FoxP3; APC-eFluor 780-conjugated antibodies against mouse Ly-6G (Gr-1); purified CD16/32 monoclonal antibody (mAb), and functional-grade antibodies against CD3. 

### 2.11. Flow Cytometry

Samples were blocked with purified FcR-blocking mAb (Thermo Fisher Scientific), washed, and suspended in PBS supplemented with 2% fetal bovine serum, 0.05% sodium azide, and a saturating concentration of fluorochrome-conjugated mAbs. The cells were analyzed using FACS Celesta (Becton Dickinson, San Jose, CA, USA).

### 2.12. Intracellular Cytokine Staining of Foxp3

Cells were labeled with primary conjugated antibodies against the surface markers CD4, CD8, and CD25, fixed with fixation and permeabilization buffer (Thermo Fisher Scientific), stained with primary conjugated antibodies against Foxp3, and analyzed using FACS Celesta. 

### 2.13. Mixed Lymphocyte Reaction (MLR) and Intracellular Cytokine Staining 

Mixed lymphocyte reaction (MLR) assays were performed as previously described [15].

Whole spleen cells from naïve mice and tumor cells from NR-S1K-grafted mice were cocultured in the presence of 0.5 μg/mL of anti-CD3 antibody for 72 h at a spleen cell–tumor cell ratio of 10:1. Four hours before the end of culture, 50 ng/mL of phorbol 12-myristate 13-acetate (Sigma-Aldrich), 500 ng/mL of ionomycin (Sigma-Aldrich), and 4 µM of monensin (Thermo Fisher Scientific) were added. After staining for surface antigens, the cells were fixed, permeabilized, and used for intracellular staining of interferon (IFN)-γ.

### 2.14. Statistical Analysis

Groups were compared using Student’s *t*-test or ANOVA using the statistical software OriginPro 2018 (OriginLab Corporation, Northampton, MA, USA), and a value of *p* < 0.05 was considered statistically significant.

## 3. Results

*3.1. mTOR Was Expressed in All Analyzed OSCC Cases*

The examined immunohistochemical localization of mTOR in the OSCC tissue samples showed that there was no apparent correlation between mTOR expression and any clinicopathological characteristic, including the tumor stage, location, and differentiation status (Table 1). 

**Figure 1:**
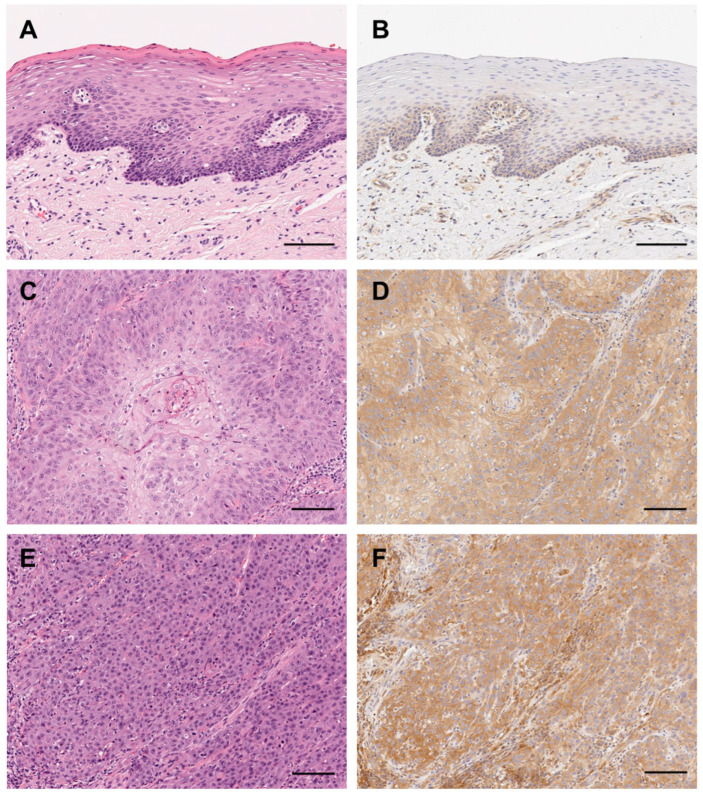
Immunohistochemical expression of mTOR in oral squamous cell carcinoma (OSCC) cases. Immunohistochemical mTOR expression profile in normal oral epithelia and OSCC tissues; normal oral epithelia (**A**,**B**), well-differentiated OSCC (**C**,**D**), and poorly differentiated OSCC (**E**,**F**). Hematoxylin and eosin (H&E) staining (**A**,**C**,**E**) and immunostaining for mTOR (**B**,**D**,**E**). Scale bars: 100 μm (**A**–**F**).

In the non-neoplastic oral mucosa including the specimens, weak mTOR signals were detected in the basal and parabasal cells of the mucosal epithelia (Figure 1A,B). The endothelial cells were also positive for mTOR. Although the staining intensity of mTOR in the OSCC cells varied among the tumors, mTOR signals were generally detected in the cytoplasm (Figure 1C–F). Keratin pearls found in the well-differentiated OSCC cells showed weaker staining than those in the less-keratinized tumor cells (Figure 1D,F).

### 3.2. Rapamycin Exerted a Direct Antitumor Effect in OSCC Cells

**Figure 2:**
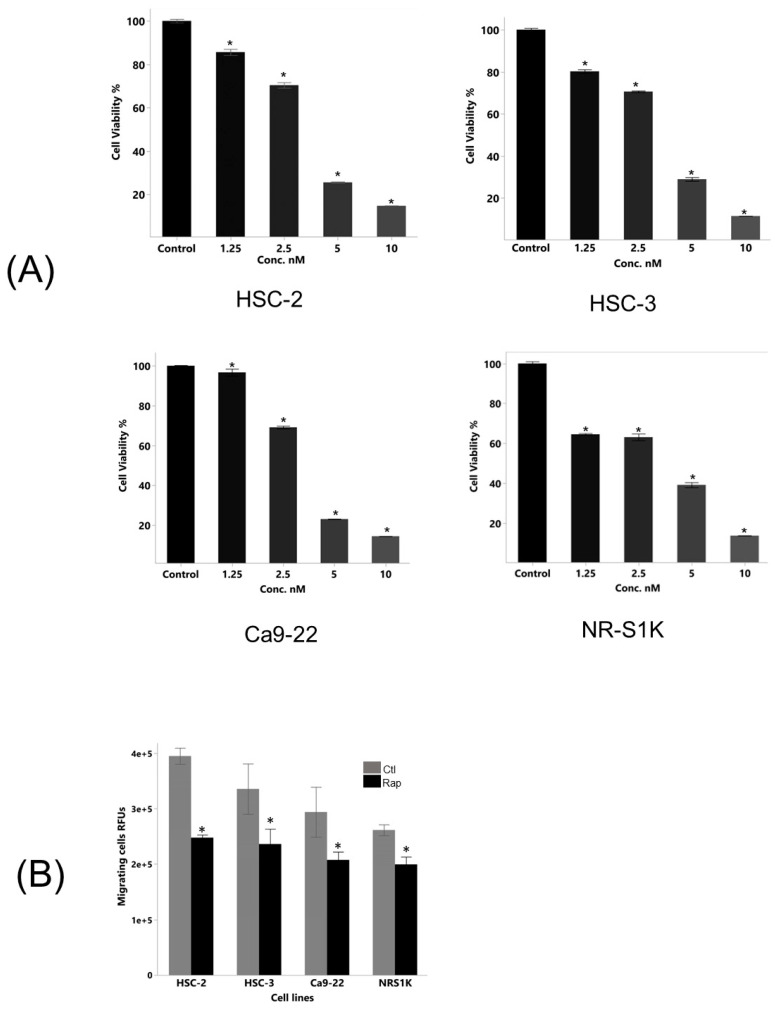

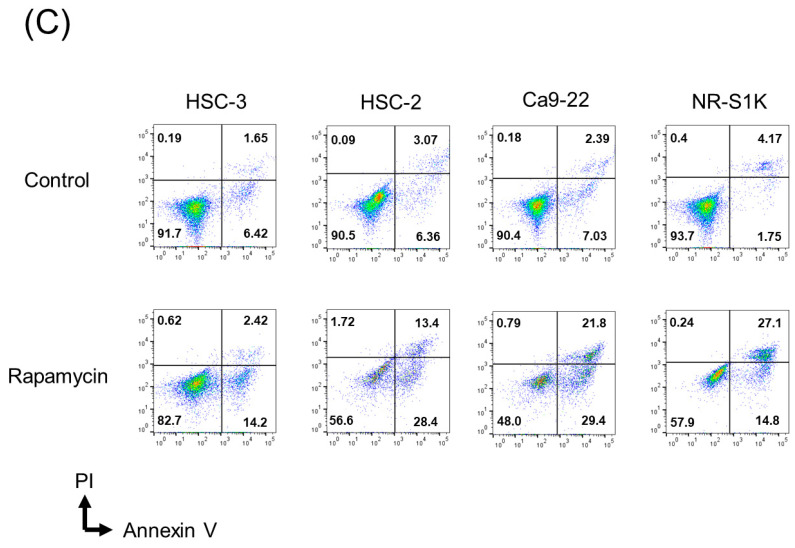
mTOR signal contributed to the proliferative and migratory activities of oral squamous cell carcinoma (OSCC) cells. (**A**) OSCC cells were cultured in the presence or absence of various concentrations of rapamycin for 72 h, and proliferative activity of cells was measured by the WST-1 cell proliferation assay. The results are presented as mean ± standard deviation from sextuplicate determinations. An asterisk indicates a significant difference between two groups (*p* < 0.05). (**B**) OSCC cells were cultured in the presence or absence of rapamycin for 48 h, and the migratory activity of cells treated with or without rapamycin was compared. The results are presented as mean ± standard deviation from quadruplicate determinations. An asterisk indicates a significant difference between two groups. (*p* < 0.02). (**C**) OSCC cells were cultured in the presence or absence of rapamycin for 48 h and had been stained with propidium iodide (PI) and annexin V for quantification.

We investigated the alteration of the viability of the OSCC cells due to rapamycin treatment. As shown in Figure 2A, the viability of the OSCC cells was significantly reduced after the rapamycin treatment in a dose-dependent manner. 

We next investigated the alteration of the migratory activity of the OSCC cells due to the rapamycin treatment. As shown in Figure 2B, the migratory activity of the OSCC cells with the rapamycin treatment was significantly lower than that of the non-treated cells.

The rapamycin treatment also significantly increased the proportion of apoptotic OSCC cells (Figure 2C).

Overall, these data provide strong evidence supporting the contribution of mTOR signaling to the progression of OSCC cells and the tumoricidal efficacy of rapamycin via modulating these cellular processes, although off-target effects cannot be ruled out.

### 3.3. Rapamycin Altered Surface Antigen Expression in OSCC Cells

**Figure 3:**
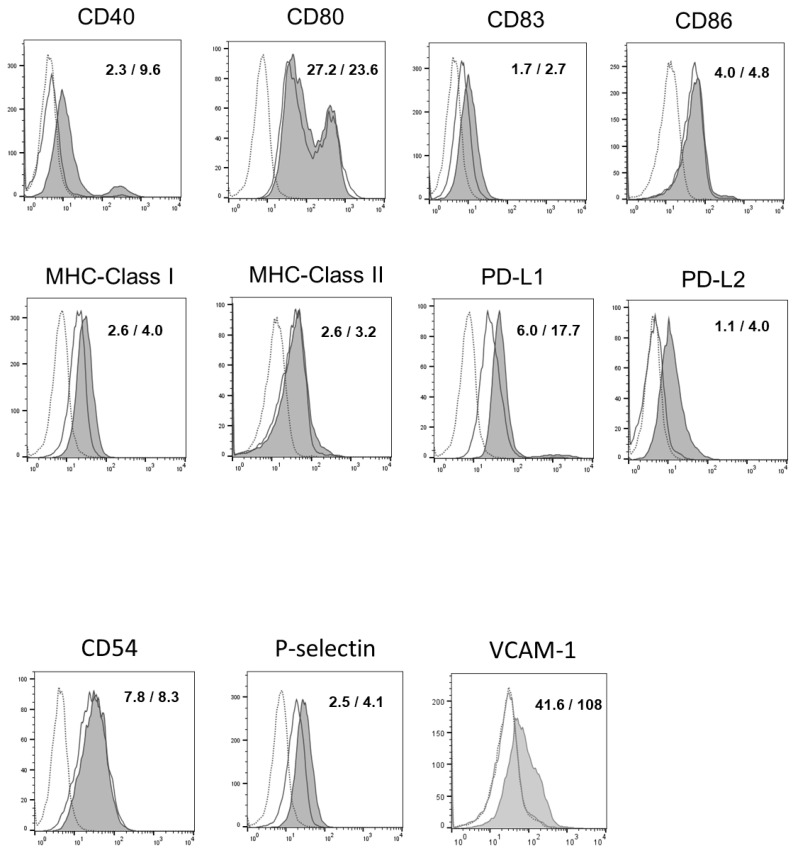
Rapamycin induced phenotypic alteration in oral squamous cell carcinoma (OSCC) cells. OSCC cells were cultured in the presence or absence of rapamycin for 72 h, and cell surface expression of various molecules was analyzed using flow cytometry. Experiments were performed in triplicate and similar results were obtained. Representative histograms from one experiment are shown. Dashed lines, non-filled solid lines, and filled solid lines indicate results for non-stained cells, non-treated cells, and rapamycin-treated cells, respectively. Numbers in each panel indicate the mean fluorescence intensity of each molecule in non-treated cells (**upper**) and rapamycin-treated cells (**lower**).

As shown in Figure 3, the OSCC cells treated with rapamycin showed increased levels of costimulatory molecules, including CD40, CD83, PD-L1, PD-L2, and MHC class I, compared to the non-treated cells. Conversely, there were no changes in the expression levels of CD80, CD86, or and MHC-class II molecules. The OSCC cells treated with rapamycin showed increased levels of P-selectin and VCAM-1, but not ICAM-1, compared to the non-treated cells (Figure 3).

### 3.4. Rapamycin Modulated the Distribution of Immune Cell Populations in In Vivo Mouse OSCC Model

**Figure 4:**
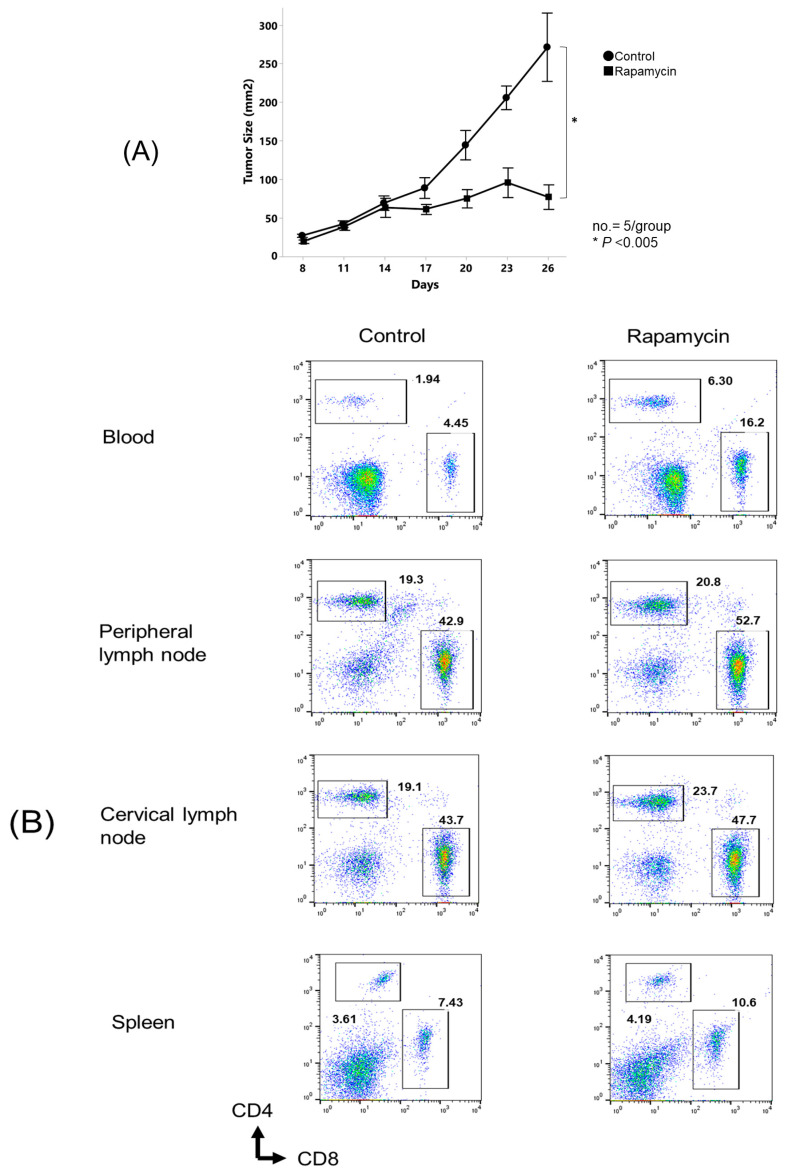

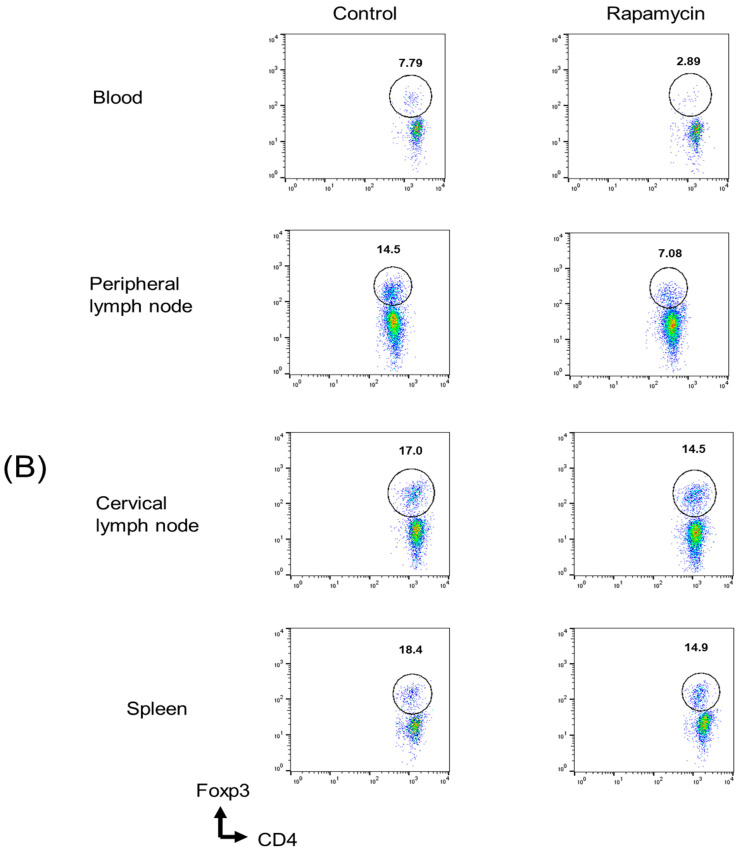

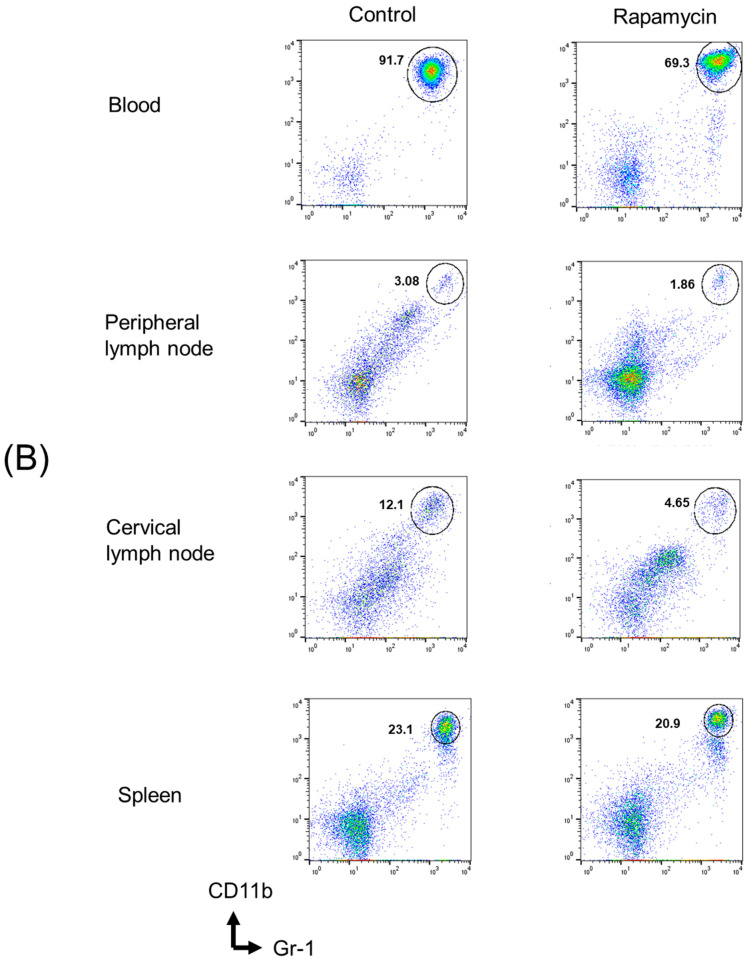

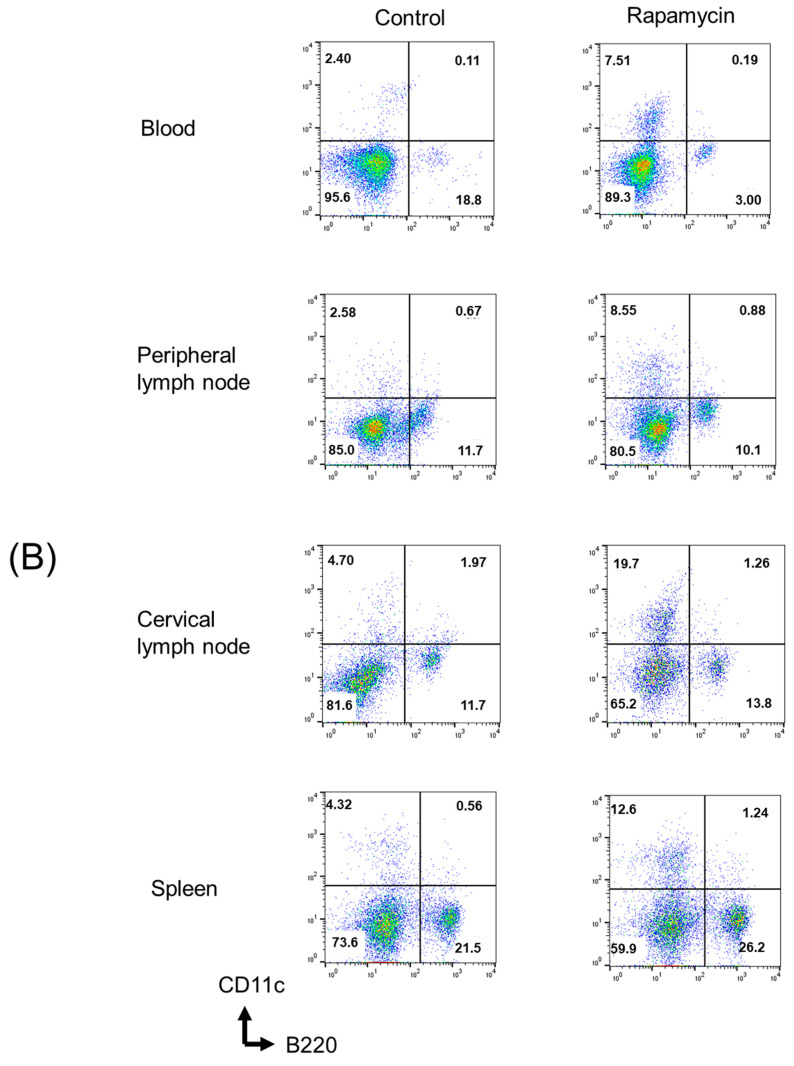

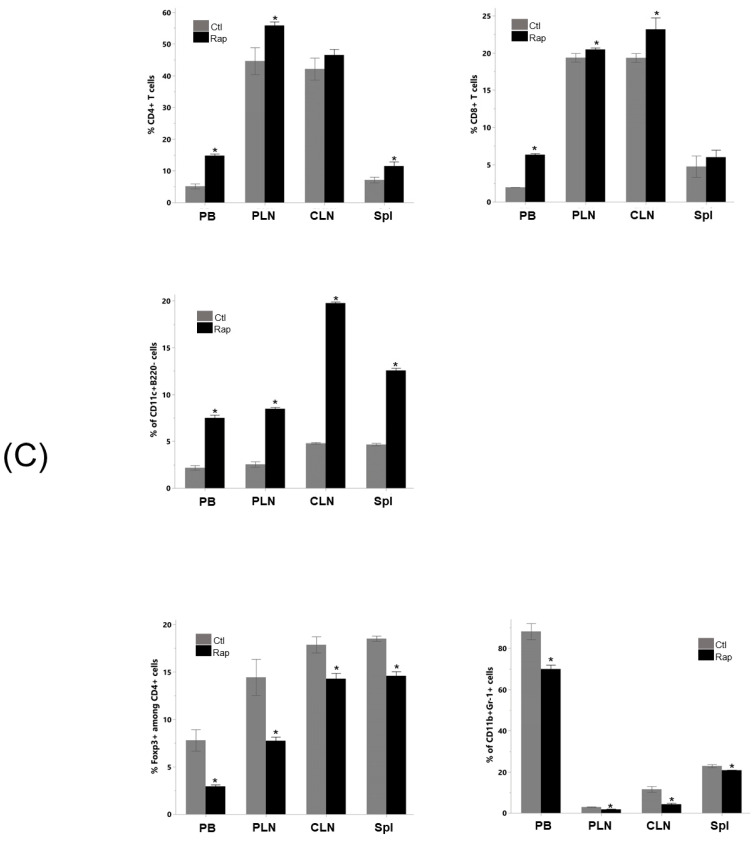
In vivo rapamycin administration altered the proportion of immune cells in oral cancer-bearing mice. Mice were challenged with NR-S1K cells. After 14 days, mice were treated with either 2 mg/kg of rapamycin or saline every 2 days. (**A**) Tumor sizes in mice were measured at the indicated time points (*n* = 5/group); *p* < 0.05, control (non-treated) vs. rapamycin. After 12 days of treatment, peripheral blood, cervical lymph nodes, peripheral lymph nodes, and spleen were harvested and the percentages of different immune cell types in each organ in control mice or rapamycin-treated mice were determined using flow cytometry. (**B**) Representative scatter plots and (**C**) summary of these results are shown (*n* = 4/group); *p* < 0.05, control vs. rapamycin. An asterisk indicates a significant difference between two groups (*p* < 0.05). (PB: peripheral blood, PLN: peripheral lymph nodes, CLN: cervical lymph nodes, Spl: spleen).

As shown in Figure 4A, the rapamycin-treated mice showed significantly delayed tumor growth compared with the control mice. Figure 4B,C show that the total proportion of CD4^+^T cells was significantly increased in the peripheral blood, peripheral lymph nodes, and spleen, but not in the cervical lymph nodes of the rapamycin-treated mice compared with the control mice.

The total proportion of CD8^+^T cells was significantly increased in the peripheral blood, peripheral lymph nodes, and cervical lymph nodes, but not in the spleens of the rapamycin-treated mice compared to the control mice. The total proportion of dendritic cells (DCs) was significantly increased in the peripheral blood, peripheral lymph nodes, cervical lymph nodes, and spleens of the rapamycin-treated mice compared to the control mice. Figure 4B,C also show that the total proportions of myeloid-derived suppressor cells (MDSCs) and regulatory T cells (Tregs) were significantly decreased in the peripheral blood, peripheral lymph nodes, cervical lymph nodes, and spleens of the rapamycin-treated mice compared to the control mice. 

### 3.5. In Vivo Rapamycin Administration Induced Phenotypic Alterations of Dendritic Cells in OSCC Tumor-Bearing Mice

**Figure 5:**
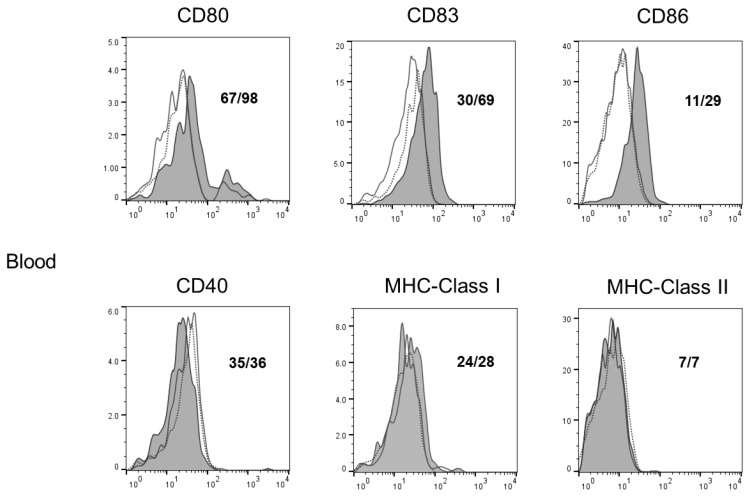

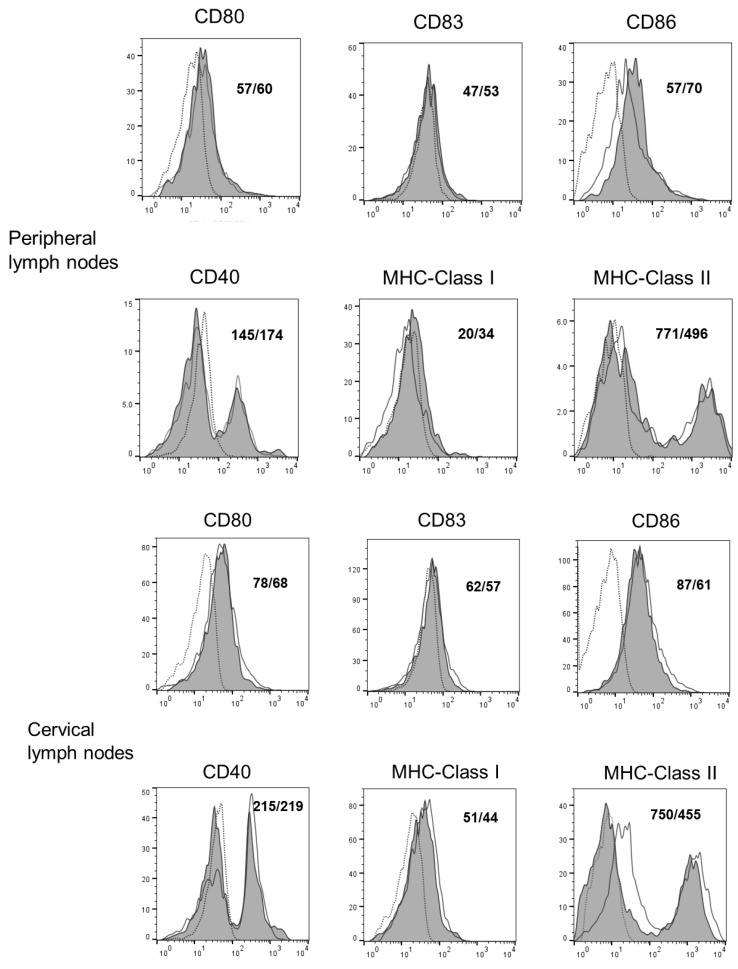

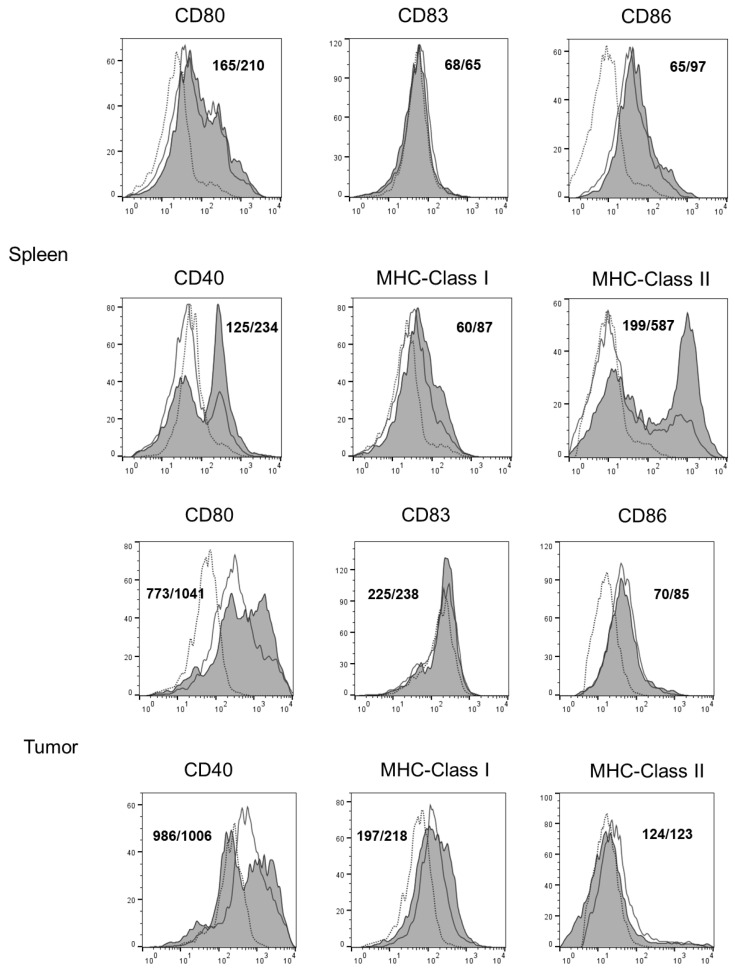
In vivo rapamycin administration induced phenotypic alterations in dendritic cells (DCs) in tumors, spleens, peripheral lymph nodes, submandibular lymph nodes, and peripheral blood cells in oral cancer-bearing mice. The phenotypic profiles of dendritic cells in NR-S1K tumor-bearing mice after rapamycin administration were analyzed. Cells were harvested from each organ of rapamycin-treated or non-treated (control) OSCC-bearing mice, and the cell surface expression of various immune accessory molecules on CD11c^+^ cells was determined using flow cytometry. Representative histograms are shown (n = 4/group). Dashed lines, unfilled solid lines, and filled solid lines indicate the results for non-stained cells, control mice, and rapamycin-treated mice, respectively. Numbers in each panel indicate the mean fluorescence intensity of each molecule in the control mice (**upper panel**) and rapamycin-treated mice (**lower panel**).

Figure 5 shows the evaluation of the surface antigen expression alterations in the tumor-bearing mice in vivo due to the rapamycin treatment. The DCs from the rapamycin-treated mice expressed higher levels of CD80, CD83, and CD86 in the peripheral blood; CD86 and MHC Class I in the peripheral lymph nodes; and CD40, CD80, CD86, MHC Class I, and MHC Class II in the spleens; and CD80 in the tumor than the dendritic cells from the control mice.

### 3.6. In Vivo Rapamycin Administration Induced Phenotypic Alterations of Tumor Cells That Facilitated Antitumor T Cell Responses 

**Figure 6:**
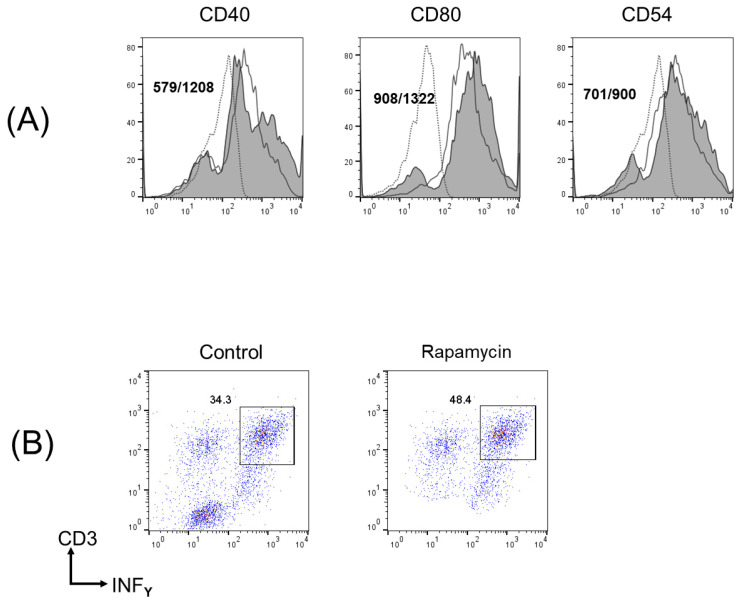
In vivo rapamycin administration induced phenotypic alterations in tumor cells that facilitate T cell responses. (**A**) Phenotypic alterations of tumor cells in oral cancer-bearing mice after rapamycin administration were analyzed. Tumor cells were harvested from rapamycin-treated or non-treated (control) OSCC-bearing mice and cell surface expression of various immune accessory molecules were determined using flow cytometry. Representative histograms are shown (n = 4/group); *p* < 0.05, control vs. rapamycin. Dashed lines, non-filled solid lines, and filled solid lines indicate results for non-stained cells, cells from control mice, and cells from rapamycin-treated mice, respectively. Numbers in each panel indicate the mean fluorescence intensity of each molecule in control mice (**upper**) and rapamycin-treated mice (**lower**). (**B**) T cell responses from OSCC cells from in vivo rapamycin-treated mice were measured in vitro. Spleen cells (1 × 10^5^) from naïve mice and tumor cells (1 × 10^4^) from rapamycin-treated or control mice were cocultured in a 96-well U-bottom culture plate in the presence of 0.5 μg/mL anti-CD3 for 72 h. Cells were restimulated with 50 ng/mL PMA, 500 ng/mL ionomycin, and 4 μM monensin 4 h before the end of culture. Interferon (IFN)-γ-producing T cells were determined using flow cytometry. Experiments were performed in triplicate and similar results were obtained. Representative scatter plots from one experiment are shown.

The tumor cells from the rapamycin-treated mice expressed higher levels of CD40, CD80, and ICAM-1 than those from the control mice (Figure 6A). In contrast, there were no changes in the expression of other immune accessory molecules, including CD86, CD83, PD-L1, PD-L2, MHC class I, and MHC class II. Moreover, we assessed the expression of adhesion molecules, including ICAM-1, VCAM-1, and P-selectin and found no changes in the expression of these molecules.

We then evaluated whether the in vivo rapamycin-treated OSCC cells could facilitate a tumor-specific T cell response. Figure 6B shows that the IFN-γ-producing CD3^+^ T cells were significantly increased when cultured with NR-S1K cells from the in vivo rapamycin-treated mice compared with NR-S1K cells from the control mice, suggesting that the tumor cells treated with rapamycin had the capacity to stimulate T cells.

## 4. Discussion

The abundant evidence of cancer heterogeneity necessitates the development of personalized medicine and targeted therapies. The dysregulation of multiple cell-signaling cascades is a hallmark of cancer; hence, the identification of potential targets among these cellular signaling pathways is urgently needed for cancer treatment.

Many studies have suggested that the constitutive activation of Akt and consequently mTOR signaling is observed in many cancers; this AKT/mTOR signaling axis is central to the proliferation, invasion, angiogenesis, migration, and epithelial-to-mesenchymal transition (EMT) of cancer cells and is indispensable in the cellular mechanism of resistance to chemotherapy and radiotherapy [8,9,10,18,19,20,21,22], whereas little is known about the direct antitumor effect of rapamycin in OSCC cells. 

In the present study, we demonstrated that the inhibition of mTOR signaling via rapamycin caused antitumor effects in a mouse model of oral cancer. Particularly, these effects were rapamycin-induced phenotypic alterations in the tumor cells and the maturation of DCs, which facilitate antitumor immune responses. Therefore, these results strongly suggest that rapamycin has direct antitumor and immunomodulatory effects on oral cancer. 

The emerging role of the immune system in cancer and its potential as a novel alternative to cancer treatment has received increased attention. In particular, the emergence of immune checkpoint inhibitors (ICIs) has revolutionized the treatment of recurrent and/or metastatic oral cancer, resulting in a better overall survival rate than from conventional therapies [5]. Moreover, accumulating evidence has shown that chemotherapy, which is generally considered immunosuppressive, activates the host immune system, resulting in enhanced antitumor effects. Certain types of molecular-targeted therapies used in combination with other chemotherapeutic agents have also been reported to enhance the host immune system. Therefore, there may be synergistic effects between immunotherapy and other modalities, including chemotherapy and molecular-targeted therapy [6].

Recent studies have focused on the regulatory function of mTOR signaling in immunological features in several pathological conditions, including cancer.

Previous studies have revealed the effects of mTOR signaling modulation in multiple immune cells. The inhibition of mTOR via rapamycin promotes CD8^+^ T cell differentiation [23,24,25]. Rapamycin has also been demonstrated to affect B cell and CD4^+^ T cell responses in a mouse model of viral infection. Rapamycin treatment during acute infection with lymphocytic choriomeningitis virus suppressed virus-specific B cell responses by inhibiting the proliferation of germinal center (GC) B cells [26]. The in vitro stimulation of rapamycin-treated human monocyte-derived DCs with proinflammatory cytokines induced a mature type 1 facilitating phenotype, consisting of elevated levels of various costimulatory molecules and augmented interleukin (IL)-12 production but decreased IL-10 secretion, although rapamycin treatment itself interfered with DC maturation [27]. The modulation of the mTOR signaling pathway has also been suggested to contribute to the immune features of the tumor microenvironment and facilitate antitumor immune responses based on preclinical and clinical data. 

The function of various types of immune components, including T cells, B cells, macrophages, and DCs can be altered via the modulation of mTOR signaling in the tumor microenvironment, suggesting a therapeutic benefit of cancer immunotherapy via targeting the mTOR signaling pathway [28,29]. Moreover, the mTOR signaling pathway may be involved in immune evasion in cancer via driving immunosuppressive features. PD-L1 expression is regulated via mTOR signaling in a mouse model of lung cancer [30]. The interaction between PD-1 and PD-L1 attenuates Akt/mTOR signaling in naïve CD4^+^ T cells and inhibits their differentiation into effector T cells [31]. In the presence of transforming growth factor-β, the PD-1-pathway-mediated downregulation of Akt/mTOR signaling induces Treg cell development, resulting in the augmentation of immunosuppressive conditions in tumor-bearing hosts [31]. A more recent study revealed that blocking the PD-1/PD-L1 pathway reduced the apoptosis of CD8^+^ T cells cocultured with a gastrointestinal stromal tumor via the activation of the PI3K/Akt/mTOR signaling pathway [32]. Tumor-associated macrophages, which are immunosuppressive cells in tumor-bearing hosts, have also been demonstrated to exhibit pro-tumoral activity via mTOR signaling activation. Furthermore, several studies have suggested the involvement of mTOR signaling in chemokine-mediated immune cell chemotaxis during tumor progression [33,34,35].

Taken together, Akt/mTOR signaling is important in direct tumor progression and in human cellular and humoral immunity via the modulation of multiple immune cells. 

Targeting the mTOR signaling pathway in cancer therapy may have a strong therapeutic benefit, directly inhibiting tumor progression and simultaneously enhancing antitumor immune responses. A recent study has suggested the synergistic effects of the combination of immunotherapy drugs and mTOR inhibitors in cancer treatment [36].

Although targeting mTOR signaling may be expected to exhibit potent antitumor immunomodulatory effects in cancer therapy, one of the critical issues is how rapamycin can be utilized to maximize treatment benefits and improve therapeutic efficacy in novel combinations.

The response rate to immunotherapy using ICIs among patients with advanced oral cancer remains still low [37]. As numerous patients display primary or acquired resistance, prognostic indicators for patients receiving immunotherapy need to be urgently identified along with the causes of resistance and strategies to overcome them [38]. The accumulation of immune regulatory cells, such as Tregs and MDSCs in the immune system of tumor-bearing hosts has been suggested as one of the reasons for the lower response to ICIs [39,40,41]. Therefore, it would be important to understand how immune regulatory cells influence the response to ICIs and how these cell populations should be targeted in cancer treatment. In our previous study, the impaired proliferative capacity of T cells following TCR stimulation and an increased number of immune regulatory cells, including Tregs and MDSCs in oral cancer-bearing mice, was observed compared to that in control mice [15,16]. In the present study, rapamycin shows an inhibitory effect on immune regulatory cells in oral cancer-bearing hosts. Therefore, pharmacological mTOR inhibition may ameliorate the poor response to immunotherapy using ICIs in oral cancer. 

## 5. Conclusions

Overall, our data suggest that mTOR signaling plays an indispensable role in the proliferation, migration, and survival of oral cancer cells and regulates various immunological functions in oral tumor-bearing hosts. Because the synergistic effect of chemo-immunotherapy has generated much interest as a new therapeutic strategy, mTOR signaling may be a potential therapeutic target for cancer therapy as a central regulator for tumor progression and immune evasion in tumor-bearing hosts. Further studies are necessary to elucidate an effective and safe clinical setting for mTOR inhibitors and to develop a more personalized medicine for oral cancer treatment.

## Figures and Tables

**Table 1:** Correlation between mTOR immune reactivity and clinicopathological variables in 27 patients with oral squamous cell carcinoma.

		mTOR (H Score)	
	n	Median (Interquartile Range)	*p*-Values
**Age, years**			0.29
<65	11	131.80 (97.00–161.40)	
≧65	16	167.70 (100.43–192.90)	
**Sex**			0.54
Male	15	153.80 (97.00–221.00)	
Female	12	142.35 (108.33–180.65)	
**Primary site**			0.71
Tongue	10	135.15 (96.28–162.73)	
Mandibular gingiva	7	161.40 (129.10–193.10)	
Maxillary gingiva	5	183.00 (96.25–232.20)	
Buccal mucosa	3	192.30 (92.60–236.30)	
Floor of mouth	2	135.30 (97.00–173.60)	
**Differentiation**			0.63
Well	8	115.25 (81.88–186.15)	
Moderate	9	138.50 (94.80–228.65)	
Poor	10	163.70 (128.03–190.20)	
**Tumor stage**			0.65
I	5	129.10 (57.75–216.90)	
II	8	161.6 (151.78–187.63)	
III	7	101.40 (85.90–189.50)	
IV	7	133.60 (97.00–193.10)	
**Smoking**			0.98
Absence	14	147.50 (112.88–190.20	
Presence	13	151.10 (94.80–207.05)	
**Drinking**			0.22
Absence	13	161.80 (124.25–193.35)	
Presence	14	133.80 (90.93–185.53)	

## Data Availability

Any personal or patient data are unavailable due to privacy or ethical restrictions. All other data are available from the corresponding author upon reasonable request.
